# Genetics of reproductive performance across Porcine Reproductive and Respiratory Syndrome (PRRS) outbreak phases in purebred and crossbred sows

**DOI:** 10.1186/s12711-025-01011-y

**Published:** 2025-10-30

**Authors:** Leticia F. de Oliveira, Jenelle Dunkelberger, Claudia A. Sevillano, Saranya Arirangan, Robbee Wedow, Matthew Tegtmeyer, Mitchell Tuinstra, Luiz F. Brito

**Affiliations:** 1 Department of Animal Sciences, Purdue University, West Lafayette, IN USA; 2 Topigs Norsvin USA, Bloomington, Minnesota USA; 3 Topigs Norsvin Research Center, ’s-Hertogenbosch, The Netherlands; 4 Sociology Department, Purdue University, West Lafayette, IN USA; 5 Department of Medical and Molecular Genetics, Indiana University School of Medicine, Indianapolis, IN USA; 6 Department of Biological Sciences, Purdue University, West Lafayette, IN USA; 7 Department of Agronomy, Purdue University, West Lafayette, IN USA

## Abstract

**Background:**

Porcine Reproductive and Respiratory Syndrome (PRRS) is a major challenge for the worldwide pig industry. Therefore, genetic selection for enhanced disease resilience is a priority for pig breeding programs. The objectives of this study were to evaluate genetic variation in reproductive performance during a PRRS outbreak and to assess the impact of selecting for enhanced reproductive performance using data collected under non-challenged conditions on reproductive performance in a PRRS challenged environment. These objectives were addressed by identifying natural PRRS outbreak periods from longitudinal performance data and estimating genetic parameters for reproductive performance traits, before, during, and after a PRRS outbreak, using data collected from purebred and crossbred sows on multiplier farms.

**Results:**

During PRRS outbreaks, the number of piglets born alive decreased, while the number of stillborn and mummified piglets increased for both purebred and crossbred sows. Additive genetic variance and heritability estimates for reproductive performance traits varied by phase. For most traits, additive genetic variance was highest during the outbreak. Estimates of genetic correlations between a given trait measured across phases ranged from 0.09 to 0.99, but were > 0.3 for most traits. In general, estimates of genetic correlations were greatest between a given trait before and after an outbreak. Results also indicated reranking of animals based on estimated breeding values across outbreak phases, with Spearman correlations below 0.50 for most traits and low proportion of top-ranking animals retained across phases.

**Conclusions:**

PRRS outbreak periods can be detected by evaluating phenotypic variation in reproductive performance from longitudinal data. Reproductive performance is heritable, whether evaluated before, during, or after a PRRS outbreak, but estimates varied by phase. Favorable moderate-to-high genetic correlations were estimated for reproductive performance traits measured before vs. during a PRRS outbreak, suggesting that selection for improved reproductive performance under non-challenged conditions is also expected to improve reproductive performance under PRRS challenge conditions. However, the estimates of genetic correlation for most of the reproductive traits indicated genotype-by-environment interactions between the PRRS-free and challenge conditions. Therefore, incorporating data collected under PRRS challenge will capture additional genetic variation in PRRS resilience and, ultimately, aid in selecting sows with increased PRRS resilience.

**Supplementary Information:**

The online version contains supplementary material available at 10.1186/s12711-025-01011-y.

## Background

Porcine Reproductive and Respiratory Syndrome (PRRS) is a highly contagious viral disease in pigs that has severely impacted the global swine industry over the past few decades. Osemeke et al. [1] estimated annual productivity losses due to PRRSV at US$1.2 billion based on data collected between 2016 and 2020, which was a significant increase from the US$663.91 million reported by Holtkamp et al. [2] based on data collected between 2005 and 2010. For this analysis, US$380.82 million of total losses were attributed to the breeding herd [1]. First identified in North America in 1987 and in Western Europe in 1990 [3], PRRS is caused by the PRRS virus (PRRSV), which has since become a global challenge for pig production. PRRSV strains are grouped into *Betaarterivirus suid 1* (a.k.a. European PRRSV-1) and *Betaarterivirus suid 2* (a.k.a. North American PRRSV-2) [4, 5], with numerous isolates per strain. Rapid evolution of the PRRSV reduces efficacy of disease control strategies, such as vaccination and biosecurity practices [6].

PRRSV-infection leads to reproductive and respiratory issues in pigs of all ages but has particularly devastating effects on sows and piglets when sows are infected during late gestation. PRRSV-infection during late gestation may result in reproductive failure, including abortion, stillbirth, or the birth of weak piglets with increased preweaning mortality [7–9]. In piglets, PRRSV generally causes respiratory disease symptoms, often accompanied by secondary bacterial and viral infections, reduced growth rate, and increased mortality [8, 9]. During an outbreak, the PRRSV can spread throughout a herd in as little as three to seven days, depending on the size and composition of the group [9]. This is known as the acute challenge phase and affects pigs across all ages and stages of production. The next phase is characterized by reproductive failure, with particularly detrimental effects for sows that are viremic during the third trimester of gestation, and elevated preweaning mortality, which can persist for one to four months [9]. Although sows may begin to recover during this period, PRRSV often continues to circulate within the herd [9].

Improving disease resilience, which can be defined as the ability of an individual to be minimally affected by disease or to rapidly recover from it [10], through genetic selection is a key strategy for ensuring the long-term sustainability of the livestock industry [10–12]. When studying the genetic basis of resilience to a particular disease, experimental infection models are useful for minimizing the influence of confounding factors, such as nutrition and environmental variation, and enable collection of repeated phenotypic measurements and accurate assessment of virus-related traits, including viral load, peak viremia, and clearance time [13]. However, studying the genetic control of host response to infection typically requires large numbers of infected animals, which is practically and economically challenging, especially if the objective is to assess genetic variation in response to PRRS in pregnant sows [13, 14]. Many experimental PRRSV challenge studies have been conducted in young pigs [15–18], leading to the identification of multiple genomic regions associated with immune response and resilience, such as the association of the SNP WUR0000125 on *Sus scrofa* chromosome 4 (SSC4) with PRRS viral load and growth rate under an experimental PRRSV challenge [15, 17, 18]. Sanglard et al. [19] investigated the genomic basis of antibody response to PRRSV vaccination and its relationship with reproductive performance in non-infected commercial sows. This approach is supported by the fact that PRRSV vaccination with a modified live virus stimulates immune evasion mechanisms, similar to those triggered by natural infection [20].

Alternatively, analyzing data from natural outbreaks presents a cost-effective opportunity to assess disease resilience using large datasets, since reproductive performance data are routinely recorded on farms. Several studies have evaluated genetic variation in reproductive performance before, during, or after a PRRS outbreak [21–24]. However, most of these studies focused exclusively on either purebred [21–23] or crossbred sows [24]. Therefore, combined reproductive performance of purebred and crossbred sows measured before, during, and after PRRS challenge has not yet been evaluated. Understanding potential differences in resilience between purebred and crossbred populations could help optimize breeding strategies (e.g., inclusion of crossbred phenotypes in purebred genomic evaluations) and crossbreeding programs to maintain productivity during disease outbreaks. In this context, the main objectives of this study were to: (1) identify PRRS outbreak periods based on phenotypic variation in longitudinal reproductive performance data (collected on purebred and crossbred sows); (2) estimate genetic parameters for reproductive performance across the outbreak phases, including before, during, and after a PRRS outbreak (considering purebred and crossbred sows analyzed either separately or together); and (3) to evaluate potential genotype-by-environment interactions (GxE) across PRRS outbreak phases.

## Methods

### Data

Reproductive performance data collected on Topigs Norsvin Genetics (‘s-Hertogenbosch, Netherlands) farms were used for this study, including data from purebred (Large White) and crossbred (Landrace x Large White) sows. Data originated from three different multiplier farms located in the United States (Farms 1, 2, and 3) and one multiplier farm located in Spain (Farm 4). Farms 1 and 4 consisted of both purebred and crossbred females, and Farms 2 and 3 consisted of only purebred females. Table 1 presents a summary of the datasets used for this study. Each farm had reported only one event of PRRS outbreak. Reproductive performance data were collected according to each farm’s routine data collection process. The sows on average had 3.12 ± 1.96 recorded parities in the data set, with a minimum of 1 and maximum of 11 parities recorded per sow. The average number of farrowings per sows recorded during the outbreaks was 1.04 ± 0.19.

**Table 1:** The numbers of sows and records, and the timeframe of data collection for purebred and crossbred sows at four multiplier farms

Farm	Number of sows	Number of records	From	To
Purebred (Large White)				
Farm 1	2386	6805	25 November 2017	14 April 2024
Farm 2	2942	5113	21 May 2022	24 November 2023
Farm 3	6994	25,040	05 December 2020	12 May 2024
Farm 4	1278	5600	01 February 2015	09 May 2024
All	13,600	42,558		
Crossbred (Landrace x Large White)				
Farm 1	6289	18,598	01 January 2022	20 June 2024
Farm 4	5027	21,468	23 July 2016	30 September 2024
All	11,316	40,066		

The traits evaluated in this study included: total number of piglets born (TNB), number of piglets born alive (NBA), number of piglets born mummified (MUM), number of piglets stillborn (NSB), number of piglets born dead (NBD; the sum of MUM and NSB), number of piglets weaned (NW), proportion of piglets born alive (propBA = NBA/TNB), proportion of piglets weaned (propW = NW/NBA), gestation length (GL, in days), and the interval from weaning to first insemination in the next cycle (IWI, in days). Quality control measures consisted of removing farrowing records with a total litter size (including NBA, NSB, and MUM) of “0”, or more than three standard deviations from the population mean; and removing records for which NW was greater than NBA. Although the farms performed crossfostering, this information was not comprehensively recorded in the studied herds and not incorporated to the analyses.

Descriptive statistics of the phenotypic data after quality control are in Table 2.

**Table 2:** Descriptive statistics for phenotypes recorded on purebred and crossbred sows

Trait^a^	Number of sows	Number of records	Mean	Minimum	Maximum	SD^b^	CV (%)^c^
TNB	13,580	42,349	16.43	1	28	3.91	23.77
Purebred Large White							
NBA	13,580	42,349	14.38	0	27	3.94	27.41
MUM	13,580	42,345	0.86	0	22	1.94	227.12
NSB	13,580	42,349	1.19	0	23	1.71	143.50
NBD	13,580	42,345	2.05	0	24	2.74	133.89
NW	11,929	33,295	11.32	0	21	3.98	35.14
propBA	13,580	42,349	0.88	0	1	0.16	17.81
propW	11,912	33,230	0.74	0	1	0.25	33.93
GL	13,580	42,349	114.63	101	125	1.47	1.28
IWI	11,908	36,113	8.75	0	75	10.60	121.12
Crossbred (Landrace x Large White)							
TNB	11,311	39,917	15.67	1	28	4.07	26.01
NBA	11,311	39,917	14.13	0	26	3.65	25.84
MUM	11,311	39,917	0.44	0	16	1.09	250.24
NSB	11,311	39,917	0.80	0	23	1.38	173.61
NBD	11,311	39,917	1.23	0	25	1.85	150.48
NW	10,227	28,901	11.49	0	22	3.43	29.88
propBA	11,311	39,917	0.91	0	1	0.13	14.51
propW	10,216	28,853	0.75	0	1	0.23	30.11
GL	11,311	39,916	115.55	101	134	1.35	1.17
IWI	4579	18,905	6.86	0	68	6.65	96.91

^a^TNB: total number of piglets born, NBA: number of piglets born alive, MUM: number of piglets born mummified, NSB: number of piglets stillborn, NBD: number of piglets born dead, NW: number of piglets weaned, propBA: proportion of piglets born alive, propW: proportion of piglets weaned, GL: gestation length, IWI: interval from weaning to first insemination.

^b^SD: Standard deviation

^c^CV: coefficient of variation

A total of 48,459 animals (46,971 females and 1488 males) were included in the pedigree file, spanning up to 16 generations. Pedigree records were available for all purebred animals, and genotypes were available for 483 of the 501 sires of purebred sows, 27.35% of which had offspring on at least two farms. For crossbred sows, pedigree records were only available for Farm 4 animals but genotypes were available for 2054 crossbred sows (Farm 1: 1189 and Farm 4: 865). Animals were genotyped with an Illumina Geneseek (Lincoln, NE, USA) custom 25 K SNP chip containing 26,894 single nucleotide polymorphisms (SNPs). Quality control of genotype data were performed considering all animals together and consisted of removing SNPs and individuals with call rates lower than 0.90 and removing SNPs with a minor allele frequency lower than 0.05. In total, 23,301 SNPs remained after quality control and no animals were removed.

## PRRS outbreak detection

To estimate genetic variation in reproductive performance pre- vs. post-PRRSV exposure, the phenotypic data for each farm were split into three phases: before, during, and after a PRRS outbreak, based on estimates of farrowing year-week effects on reproductive performance traits that are most affected by a PRRS challenge [21, 25, 26]. The farrowing year-week estimates were obtained separately for purebred and crossbred sows based on the following model:

1 $$\:\mathbf{y}=\mathbf{X}\mathbf{b}+\mathbf{Z}\mathbf{f}\mathbf{y}\mathbf{w}+\mathbf{Z}\mathbf{s}\mathbf{o}\mathbf{w}+\mathbf{e},$$

where $\:\mathbf{y}$ is the vector of phenotypic records across farms; $\:\mathbf{X}$ is an incidence matrix of fixed effects; $\:\mathbf{b}$ is the vector of fixed effects, which comprised the effects of farm and parity (eight classes: 1, 2, 3, 4, 5, 6, 7, 8+); $\:\mathbf{Z}$ is an incidence matrix of random effects; $\:\mathbf{f}\mathbf{y}\mathbf{w}$ is the random effect of farm-year-week of the farrowing record, assuming $\:\mathbf{f}\mathbf{y}\mathbf{w}\sim\text{N}(0,\mathbf{I}{{\upsigma\:}}_{\text{f}\text{y}\text{w}}^{2}$), where $\:\mathbf{I}$ is an identity matrix and $\:{{\upsigma\:}}_{\text{f}\text{y}\text{w}}^{2}$ is the farm-year-week variance; $\:\mathbf{s}\mathbf{o}\mathbf{w}$ is the random effect of sow, assuming $\:\mathbf{s}\mathbf{o}\mathbf{w}\sim\text{N}(0,\mathbf{I}{{\upsigma\:}}_{\text{s}\text{o}\text{w}}^{2}$), where $\:{{\upsigma\:}}_{\text{s}\text{o}\text{w}}^{2}$ is the sow variance; and $\:\mathbf{e}$ is the vector of residuals, assuming $\:\mathbf{e}\sim\text{N}(0,\mathbf{I}{{\upsigma\:}}_{\text{e}}^{2}$), where $\:{\sigma\:}_{e}^{2}$ is the residual variance. The analyses were performed using the BLUPf90+ software family [27].

Estimates of farrowing year-week for a given trait were standardized by their standard deviations to make estimates comparable across traits [21] and used to identify extreme values to infer when the outbreak period occurred [24]. Due to the expected directional effects (e.g., an increase in MUM and NSB, and a decrease in NBA), one-tailed limit tests at 95% confidence were applied. The beginning of a PRRS outbreak period was defined when significant (*p*-value < 0.05) farrowing year-week estimates were observed for at least three traits and for at least three consecutive weeks. The end of the outbreak period was defined when non-significant (*p*-value ≥ 0.05) estimates of farrowing year-week were observed for at least three reproductive performance traits and for at least three consecutive weeks. Because the linear model used may not enable the identification of the early and final weeks of a disease phase when infected and recovered sows coexist [26], farrowing records that occurred within 4 weeks before the identified start and after the identified end of the outbreak, which were defined as grey periods, were removed from the subsequent analyses [26].

## Variance component estimation

Variance components were estimated based on purebred sows only, on crossbred sows only, or on both purebred and crossbred sows. Variance components and heritability estimates were obtained by separatelyanalyzing data from before and after the outbreak using model (2), which included a permanent environmental effect), and data from during the outbreak using model (3):

2 $$\:\mathbf{y}=\mathbf{X}\mathbf{b}+\mathbf{Z}\mathbf{a}+\mathbf{Z}\mathbf{p}\mathbf{e}+\mathbf{e}$$

3 $$\:\mathbf{y}=\mathbf{X}\mathbf{b}+\mathbf{Z}\mathbf{a}+\mathbf{e}$$

where $\:\mathbf{y}$ is the vector of phenotypic records for a given reproduction trait; $\:\mathbf{X}$ is the incidence matrix of fixed effects; $\:\mathbf{b}$ is the vector of fixed effects, which comprised the effects of parity (eight classes: 1, 2, 3, 4, 5, 6, 7, 8+) and contemporary group (defined as the concatenation of farm, year, and month of farrowing); $\:\mathbf{Z}$ is the incidence matrix of random additive genetic effects **a**,$\:\:\text{w}\text{i}\text{t}\text{h}\:\mathbf{a}\:\sim\:\text{N}(0,\:\mathbf{H}{{\upsigma\:}}_{\text{a}}^{2})$, where $\:{{\upsigma\:}}_{\text{a}}^{2}$ is the additive genetic variance and $\:\mathbf{H}$ is a hybrid relationship matrix combining genotyped and non-genotyped animals [28]; $\:\mathbf{p}\mathbf{e}$ is a vector of random permanent environmental effects with $\:\mathbf{p}\mathbf{e}\:\sim\:\text{N}(0,\:\mathbf{I}{{\upsigma\:}}_{\text{p}\text{e}}^{2})$, where $\:{{\upsigma\:}}_{\text{p}\text{e}}^{2}$ is the permanent environmental variance, and $\:\mathbf{I}$ is an identity matrix; and $\:\mathbf{e}$ is the vector of residuals, assuming $\:\mathbf{e}\sim\text{N}(0,\mathbf{I}{{\upsigma\:}}_{\text{e}}^{2}$) where $\:{{\upsigma\:}}_{\text{e}}^{2}$ is the residual variance. For the traits propBA and propW, logit transformations were applied before fitting a linear model to estimate variance components, ensuring that model residuals met the assumptions of normality and linearity. Bivariate models were fitted to obtain estimates of genetic correlations for each trait across phases, to evaluate the presence of GxE (estimates of genetic correlation significantly lower than 1). Variance components were estimated using the AI-REML algorithm implemented in the BLUPF90+ software [27].

Before combining the purebred and crossbred data, purebred-crossbred genetic correlations were estimated for each trait using a bivariate model, without splitting the data across PRRS outbreak phases. The same effects were fitted as described for model (2), where traits recorded on crossbreds and purebreds were assumed to be distinct, but genetically correlated, traits. When combining the purebred and crossbred data, the models fitted also included population (i.e. purebred or crossbred) as a categorical fixed effect.

## Animal reranking

Potential reranking of animals across PRRS outbreak phases was evaluated using EBVs from single-trait analyses by phase. Reranking was evaluated for purebred animals (males and females) only, as genetic improvement is made by selection at the purebred level. We assumed the following scenarios for purebred selection based on (1) records collected before a PRRS outbreak; (2) records collected during an outbreak; or (3) records collected after the outbreak. For all scenarios, we assumed that selection was based on only purebred data or on the combination of purebred and crossbred data.

We included only animals with genomic estimated breeding values (GEBV) having an accuracy greater than 0.40 for all three phases (before, during, and after the PRRS outbreak). The accuracy of GEBV was calculated as

4 $$\:accuracy=\:\sqrt{1-PEV/{\sigma\:}_{a}^{2}},$$

where $\:PEV$ is the prediction error variance and $\:{\sigma\:}_{a}^{2}$ is the estimate of the additive genetic variance of the trait [29]. Spearman rank correlations were estimated to assess consistency of GEBV rankings across outbreak phases (before vs. during, before vs. after, and during vs. after). We also evaluated the proportion of animals that were selected across phases when selecting the top 1, 5, 10, or 20%.

## Results

### PRRS outbreak detection

**Fig. 1:**
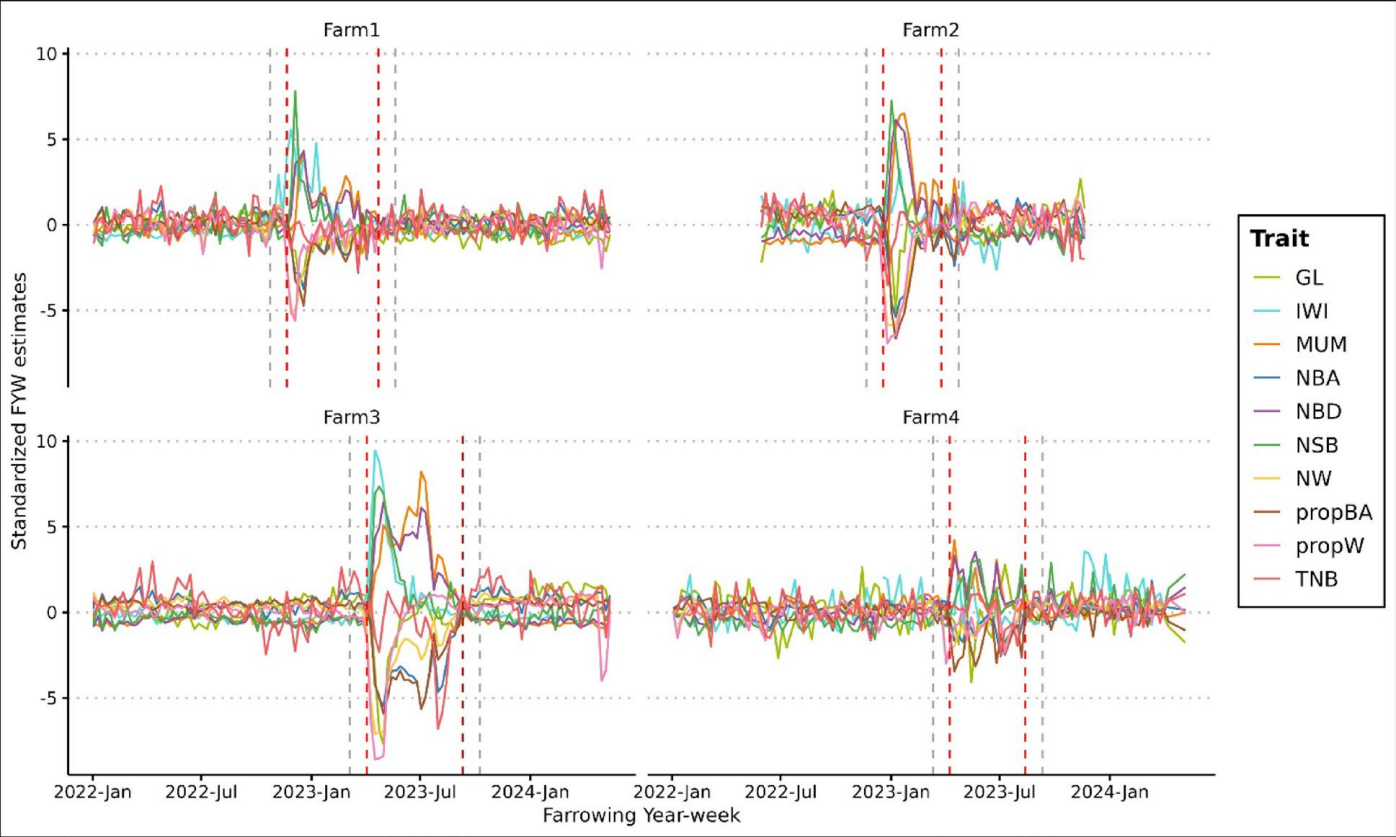
Standardized estimates of farrowing year-week effects for reproductive traits of purebred sows in four farms. Red dashed lines indicate the beginning and end of the outbreak period on each farm. Grey dashed lines indicate the grey period of four weeks prior and after the outbreak. GL: gestation length; IWI: interval from weaning to first insemination; MUM: number of mummified piglets; NBA: number of piglets born alive; NBD: number of piglets born dead; NSB: number of piglets stillborn; NW: number of piglets weaned; propBA: proportion of piglets born alive (NBA/TNB); propW: proportion of piglets weaned (NW/NBA); TNB: total number of piglets born

**Fig. 2:**
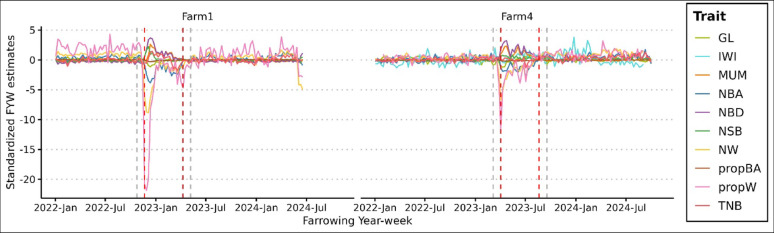
Standardized farm estimates of farrowing year-week effects for reproductive traits of crossbred sows in two farms. Red dashed lines indicate the beginning and end of the outbreak period on each farm. Grey dashed lines indicate the grey period of four weeks prior and after the outbreak. GL: gestation length; IWI: interval from weaning to first insemination; MUM: number of mummified piglets; NBA: number of piglets born alive; NBD: number of piglets born dead; NSB: number of piglets stillborn; NW: number of piglets weaned; propBA: proportion of piglets born alive (NBA/TNB); propW: proportion of piglets weaned (NW/NBA); TNB: total number of piglets born

PRRS outbreak periods were detected for each farm based on farrowing year-week estimates for NBA, MUM, NSB, NBD, NW, propBA, and propW. Figures 1 and 2 show the standardized farrowing year-week estimates for each trait within each farm, as well as the beginning and end of each outbreak period. Figure 1 presents the estimates for the purebred sows and Fig. 2 for the crossbred sows. These figures show that TNB was unaffected by the PRRS challenge for both purebred (Fig. 1) and crossbred (Fig. 2) sows. A decrease in GL and an increase in IWI was observed at the beginning of the outbreak phase for most farms and for both purebred and crossbred sows (Figs. 1 and 2). Each farm had only one PRRS outbreak during the studied period. The outbreaks in purebred populations lasted for 155, 99, 162, and 120 days in Farms 1, 2, 3, and 4, respectively, while the outbreaks in crossbred populations lasted for 140 and 141 days in Farms 1 and 4, respectively, with similar timing between purebred and crossbred populations on these farms (see Additional file 1: Table S1).

[Insert Figs. 1 and 2 here]

**Fig. 3:**
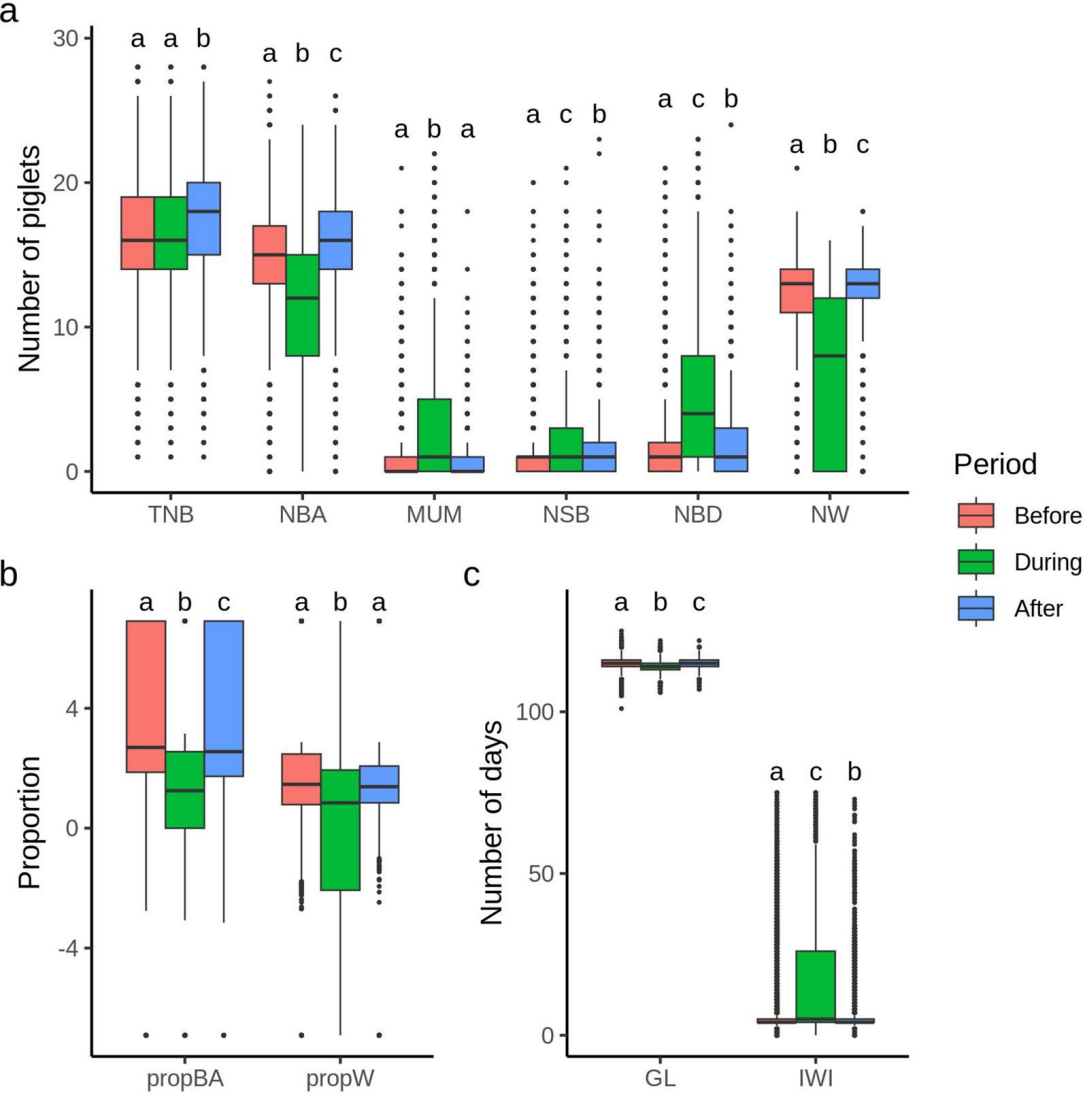
Boxplot for reproductive traits of purebred sows before, during, and after a PRRS outbreak. (**a**) Total number of piglets born (TNB), number of piglets born alive (NBA), number of mummified piglets (MUM); number of piglets stillborn (NSB); number of piglets born dead (NBD), and number of piglets weaned (NW). (**b**) Proportion of piglets born alive (propBA) and proportion of piglets weaned (propW). (**c**) Gestation length (GL) and interval from weaning to first insemination (IWI). Different letters above the boxplots indicate significant differences among periods for each trait based on Tukey’s mean comparison test (*p* < 0.05)

**Fig. 4:**
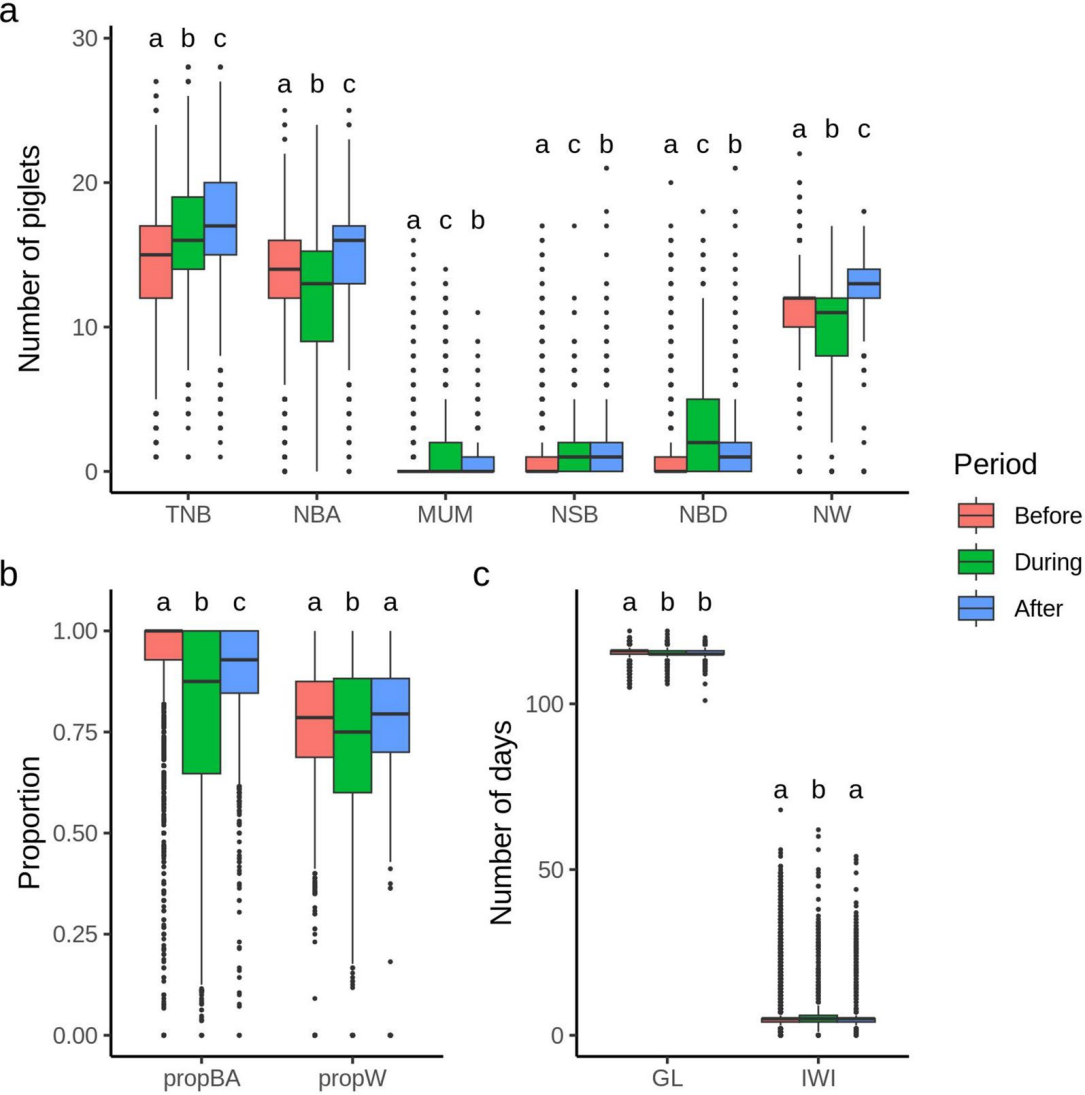
Boxplot for reproductive traits of crossbred sows before, during, and after a PRRS outbreak. (a) Total number of piglets born (TNB), number of piglets born alive (NBA), number of mummified piglets (MUM); number of piglets stillborn (NSB); number of piglets born dead (NBD), and number of piglets weaned (NW). (b) Proportion of piglets born alive (propBA) and proportion of piglets weaned (propW). (c) Gestation length (GL) and interval from weaning to first insemination (IWI). Different letters above the boxplots indicate significant differences among periods for each trait based on Tukey’s mean comparison test (*p* < 0.05)

Figures 3 and 4 show boxplots of reproductive performance traits for purebred and crossbred sows, respectively, before, during, and after the PRRS outbreak. These figures demonstrate the impact of the PRRS outbreak on these traits. Descriptive statistics for traits in each phase are provided in Additional file 2. Although PRRS did not impact TNB, an increase in MUM, NSB, and NBD was observed during each outbreak period, while NBA and NW decreased for both purebred (Fig. 3a) and crossbred (Fig. 4a) sows, as did propBA and propW (Figs. 3b and 4b). For purebred sows, GL decreased and IWI increased during the PRRS outbreak (Fig. 3c). However, for crossbred sows, no impact of PRRS exposure was observed on GL or IWI (Fig. 4c).

[Insert Figs. 3 and 4 here]

## Variance component estimation

Variance components and genetic parameters were estimated separately for reproductive performance traits for each phase (i.e., before, during, and after the PRRS outbreak) and population (i.e. purebred and crossbred populations). Table 3 presents estimates of variance components and the heritability for purebred and crossbred sows.

**Table 3:** Variance component estimates for reproductive performance traits before, during, and after PRRS outbreak

Trait^a^	Before				During			After			
	$\:{\varvec{\sigma\:}}_{\varvec{a}}^{2}$	$\:{\varvec{\sigma\:}}_{\varvec{p}\varvec{e}}^{2}$	$\:{\varvec{\sigma\:}}_{\varvec{e}}^{2}$	$\:{\varvec{h}}^{2}$	$\:{\varvec{\sigma\:}}_{\varvec{a}}^{2}$	$\:{\varvec{\sigma\:}}_{\varvec{e}}^{2}$	$\:{\varvec{h}}^{2}$	$\:{\varvec{\sigma\:}}_{\varvec{a}}^{2}$	$\:{\varvec{\sigma\:}}_{\varvec{p}\varvec{e}}^{2}$	$\:{\varvec{\sigma\:}}_{\varvec{e}}^{2}$	$\:{\varvec{h}}^{2}$
Purebred sows											
TNB	1.41 ± 0.16	1.48 ± 0.14	9.96 ± 0.11	0.11 ± 0.01	2.24 ± 0.49	15.18 ± 0.47	0.13 ± 0.03	1.91 ± 0.32	1.4 ± 0.33	10.83 ± 0.27	0.14 ± 0.02
NBA	1.01 ± 0.13	1.22 ± 0.12	9.3 ± 0.1	0.09 ± 0.01	1.91 ± 0.5	19.72 ± 0.55	0.09 ± 0.02	0.8 ± 0.19	1.79 ± 0.25	9.09 ± 0.22	0.07 ± 0.02
NSB	0.16 ± 0.02	0.17 ± 0.02	1.89 ± 0.02	0.07 ± 0.01	0.38 ± 0.1	4.46 ± 0.12	0.08 ± 0.02	0.49 ± 0.07	0.19 ± 0.07	2.32 ± 0.06	0.16 ± 0.02
MUM	0.06 ± 0.01	0.03 ± 0.01	1.11 ± 0.01	0.05 ± 0.01	1.29 ± 0.32	13.53 ± 0.36	0.09 ± 0.02	0.06 ± < 0.01	0.00 ± < 0.00	0.94 ± 0.01	0.06 ± < 0.01
NBD	0.25 ± 0.03	0.27 ± 0.03	3.2 ± 0.03	0.07 ± 0.01	1.52 ± 0.41	18.88 ± 0.49	0.07 ± 0.02	0.72 ± 0.11	0.14 ± 0.1	3.41 ± 0.08	0.17 ± 0.02
NW	0.59 ± 0.12	1.81 ± 0.14	8.19 ± 0.11	0.06 ± 0.01	1.22 ± 0.37	14.77 ± 0.43	0.08 ± 0.02	1.49 ± 0.29	0.82 ± 0.3	7.17 ± 0.23	0.16 ± 0.03
propBA	1.69 ± 0.25	1.42 ± 0.27	29.35 ± 0.31	0.05 ± 0.01	1.22 ± 0.45	25.54 ± 0.63	0.05 ± 0.02	2.37 ± 0.49	1.04 ± 0.61	24.96 ± 0.6	0.08 ± 0.02
propW	0.002 ± < 0.01	0.004 ± 0.001	0.04 ± 0.001	0.04 ± 0.01	0.003 ± 0.001	0.07 ± 0.002	0.05 ± 0.02	0.65 ± 0.30	1.98 ± 15	20.24 ± 0.64	0.03 ± 0.01
GL	0.8 ± 0.05	0.14 ± 0.03	1.02 ± 0.01	0.41 ± 0.02	0.99 ± 0.13	2.29 ± 0.10	0.30 ± 0.04	0.74 ± 0.07	0.19 ± 0.05	0.72 ± 0.02	0.45 ± 0.03
IWI	2.67 ± 0.61	11.6 ± 0.9	60.47 ± 0.79	0.04 ± 0.01	4.19 ± 2.17	165.07 ± 3.89	0.02 ± 0.01	0.97 ± 0.54	2.05 ± 1.33	41.72 ± 1.43	0.02 ± 0.01
Crossbred sows											
TNB	1.50 ± 0.23	1.05 ± 0.18	10.40 ± 0.11	0.12 ± 0.01	6.26 ± 0.92	12.43 ± 0.79	0.34 ± 0.05	2.00 ± 0.35	2.17 ± 0.35	10.52 ± 0.19	0.14 ± 0.02
NBA	0.55 ± 0.12	1.21 ± 0.12	9.72 ± 0.10	0.05 ± 0.01	3.55 ± 0.84	16.21 ± 0.83	0.18 ± 0.04	1.31 ± 0.26	1.70 ± 0.27	8.44 ± 0.15	0.11 ± 0.02
NSB	0.04 ± 0.01	0.07 ± 0.01	1.29 ± 0.01	0.03 ± 0.01	0.95 ± 0.18	2.47 ± 0.16	0.28 ± 0.05	0.09 ± 0.03	0.33 ± 0.04	1.80 ± 0.03	0.04 ± 0.02
MUM	0.06 ± < 0.01	0.0001 ± < 0.01	0.52 ± < 0.01	0.10 ± < 0.01	0.75 ± 0.19	5.22 ± 0.22	0.13 ± 0.03	0.06 ± 0.01	0.03 ± 0.02	0.65 ± 0.01	0.08 ± 0.02
NBD	0.14 ± 0.03	0.07 ± 0.02	1.90 ± 0.02	0.07 ± 0.01	1.15 ± 0.34	8.53 ± 0.38	0.12 ± 0.03	0.21 ± 0.06	0.41 ± 0.06	2.51 ± 0.04	0.07 ± 0.02
NW	3.90 ± 0.05	0.0001 ± 0	4.53 ± 0.04	0.46 ± 0.004	2.06 ± 0.72	12.95 ± 0.76	0.14 ± 0.05	6.59 ± 0.08	0.0001 ± < 0.01	8.08 ± 0.11	0.45 ± < 0.01
propBA	1.07 ± 0.27	0.91 ± 0.27	31.26 ± 0.33	0.04 ± 0.01	2.29 ± 1.09	31.08 ± 1.30	0.06 ± 0.03	1.35 ± 0.49	2.80 ± 0.58	27.26 ± 0.48	0.09 ± 0.02
propW	2.71 ± 0.04	0.0001 ± < 0.01	22.78 ± 0.21	0.40 ± < 0.01	4.13 ± 0.64	37.03 ± 1.25	0.10 ± 0.05	5.27 ± 1.13	1.19 ± 1.10	27.09 ± 0.64	0.33 ± 0.02
GL	0.60 ± 0.05	0.13 ± 0.03	1.02 ± 0.01	0.34 ± 0.02	0.76 ± 0.11	1.93 ± 0.10	0.28 ± 0.04	0.44 ± 0.05	0.59 ± 0.05	0.55 ± 0.01	0.28 ± 0.03
IWI	0.61 ± 0.25	2.47 ± 0.35	36.81 ± 0.47	0.02 ± 0.01	2.22 ± 2.62	65.21 ± 4.17	0.03 ± 0.04	0.90 ± 0.01	0.0001 ± < 0.01	40.83 ± 0.55	0.02 ± < 0.01

$\:{\sigma\:}_{a}^{2}$: genetic variance estimate.

$\:{\sigma\:}_{pe}^{2}$: permanent environmental variance estimate.

$\:{\sigma\:}_{e}^{2}$: residual variance estimate.

$$\:{\text{h}}^{2}$$: heritability estimate

^a^TNB: total number of piglets born, NBA: number of piglets born alive, MUM: number of piglets born mummified, NSB: number of piglets stillborn, NBD: number of piglets born dead, NW: number of piglets weaned, propBA: proportion of piglets born alive, propW: proportion of piglets weaned, GL: gestation length, IWI: interval weaning to first insemination.

^b^propBA and propW were logit-transformed.

## Purebreds

For purebred sows, when comparing estimates of variance components across the outbreak phases, we observed that additive genetic variances were higher during and after the PRRS outbreaks than during the outbreak phases. The residual variances after the outbreak were lower than during the outbreak phase for NSB, NBD, NW, and propBA, which resulted in higher heritability estimates for the phase after the outbreak (Table 3). Heritability estimates for MUM and propW were highest during the outbreak, where purebred sows had greater genetic variability during the outbreak, with higher variance component and heritability estimates compared to the periods before and after the outbreak (Table 3).

Table 4 presents estimates of genetic correlations for a given reproductive performance trait across phases for purebred sows. Strong, positive correlations (from 0.89 to 0.99) were estimated between phases for TNB, NSB, and GL. For NBA, MUM, NBD, NW, and propBA, estimates were 0.02 to 0.31 units higher for genetic correlations between a trait measured before/after a PRRS break, than before/during or during/after the PRRS outbreak. The lowest genetic correlations (0.09 and 0.28, for propW and IWI, respectively) were estimated between traits measured before/during an outbreak. Standard errors were ≤ 0.25 for all traits, except for IWI, for which the standard error ranged from 0.26 to 0.85.

**Table 4:** Estimates of genetic correlations disease phases (before vs. during vs. after the PRRS outbreak) for reproductive performance traits in purebred sows

Trait^a^	Before vs. during	Before vs. after	During vs. after
TNB	0.99 ± 0.04	0.94 ± 0.03	0.98 ± 0.03
NBA	0.72 ± 0.10	0.95 ± 0.05	0.78 ± 0.10
NSB	0.94 ± 0.01	0.89 ± 0.04	0.92 ± 0.04
MUM	0.83 ± 0.11	0.85 ± 0.07	0.65 ± 0.12
NBD	0.87 ± 0.01	0.89 ± 0.04	0.73 ± 0.03
NW	0.51 ± 0.21	0.69 ± 0.11	0.47 ± 0.18
propBA^b^	0.64 ± 0.24	0.95 ± 0.06	0.93 ± 0.25
propW^b^	0.09 ± 0.07	0.48 ± 0.16	0.82 ± 0.06
GL	0.97 ± 0.02	0.97 ± 0.01	0.95 ± 0.01
IWI	0.28 ± 0.67	0.82 ± 0.26	0.85 ± 0.85

^a^TNB: total number of piglets born, NBA: number of piglets born alive, MUM: number of piglets born mummified, NSB: number of piglets stillborn, NBD: number of piglets born dead, NW: number of piglets weaned, propBA: proportion of piglets born alive, propW: proportion of piglets weaned, GL: gestation length, IWI: interval from weaning to first insemination.

^b^propBA and propW were logit-transformed.

### Crossbreds

For crossbred sows, heritability estimates for TNB, NBA, NSB, MUM, and NBD were highest during the PRRS outbreak, mostly driven by higher additive genetic variances during the outbreak than before or after the outbreak (Table 3). However, heritability estimates for NW and propW were lower during the outbreak, compared to before or after the outbreak, due to a considerable increase in the residual variance for these traits during the outbreak (Table 3).

Bivariate models for the reproductive performance traits between PRRS phases did not converge for the crossbred populations. This was likely due to missing pedigree information from one of the farms and a low proportion of genotyped animals, which limited the ability to estimate genetic relationships between animals. Animals without pedigree and no genotype information were retained in the analyses to improve estimation of fixed effects (e.g., contemporary groups), but they were assumed to be unrelated to all other animals. It is worth noting that this may have biased the heritability estimates downwards.

### Combined data

Estimates of genetic correlations for reproductive performance traits between purebreds and crossbreds, irrespective of outbreak phase, are reported in Table 5. Estimates were ≥ 0.90 for all traits, except for MUM (0.11), propW (0.55), and IWI (0.59).

**Table 5:** Estimates of the purebred-crossbred genetic correlation (r_pc_) for reproductive traits, irrespective of PRRS outbreak phase

Trait^a^	*r*_pc_
TNB	0.98 ± 0.06
NBA	0.92 ± 0.01
NSB	0.93 ± 0.01
MUM	0.11 ± 0.01
NBD	0.99 ± 0.01
NW	0.99 ± < 0.01
propBA^b^	0.77 ± 0.15
propW^b^	0.55 ± < 0.01
GL	0.94 ± 0.03
IWI	0.59 ± 0.27

^a^TNB: total number of piglets born, NBA: number of piglets born alive, MUM: number of piglets born mummified, NSB: number of piglets stillborn, NBD: number of piglets born dead, NW: number of piglets weaned, propBA: proportion of piglets born alive, propW: proportion of piglets weaned, GL: gestation length, IWI: interval from weaning to first insemination.

^b^propBA and propW were logit-transformed.

Due to the high genetic correlation estimates between traits measured on purebred vs. crossbred sows, the purebred and crossbred data were combined, and genetic parameters were re-estimated using a single-trait animal model with population (“purebred” or “crossbred”) as a fixed effect. Variance components and heritability estimates are presented in Table 6.

**Table 6:** Variance component estimates for reproductive performance traits before, during, and after PRRS outbreak for purebred and crossbred sows

Trait^a^	Before				During			After			
	$\:{\varvec{\sigma\:}}_{\varvec{a}}^{2}$	$\:{\varvec{\sigma\:}}_{\varvec{p}\varvec{e}}^{2}$	$\:{\varvec{\sigma\:}}_{\varvec{e}}^{2}$	$\:{\varvec{h}}^{2}$	$\:{\varvec{\sigma\:}}_{\varvec{a}}^{2}$	$\:{\varvec{\sigma\:}}_{\varvec{e}}^{2}$	$\:{\varvec{h}}^{2}$	$\:{\varvec{\sigma\:}}_{\varvec{a}}^{2}$	$\:{\varvec{\sigma\:}}_{\varvec{p}\varvec{e}}^{2}$	$\:{\varvec{\sigma\:}}_{\varvec{e}}^{2}$	$\:{\varvec{h}}^{2}$
TNB	1.22 ± 0.11	1.26 ± 0.1	10.21 ± 0.08	0.1 ± 0.01	2.47 ± 0.42	14.68 ± 0.4	0.14 ± 0.02	1.72 ± 0.23	1.77 ± 0.24	10.69 ± 0.2	0.12 ± 0.02
NBA	0.85 ± 0.09	1.1 ± 0.08	9.74 ± 0.08	0.07 ± 0.01	2.29 ± 0.45	18.64 ± 0.47	0.11 ± 0.02	0.89 ± 0.15	1.68 ± 0.19	9.08 ± 0.17	0.08 ± 0.01
NSB	0.12 ± 0.01	0.11 ± 0.01	1.63 ± 0.01	0.06 ± 0.01	0.38 ± 0.08	3.88 ± 0.09	0.09 ± 0.02	0.3 ± 0.04	0.3 ± 0.04	2.16 ± 0.04	0.11 ± 0.01
MUM	0.04 ± 0.01	0.03 ± 0.01	0.84 ± 0.01	0.04 ± 0.01	1.18 ± 0.24	11.81 ± 0.28	0.09 ± 0.02	0.06 ± 0.01	0.02 ± 0.01	0.81 ± 0.01	0.07 ± 0.01
NBD	0.2 ± 0.02	0.19 ± 0.02	2.62 ± 0.02	0.07 ± 0.01	1.45 ± 0.32	16.42 ± 0.38	0.08 ± 0.02	0.47 ± 0.06	0.33 ± 0.06	3.1 ± 0.06	0.12 ± 0.02
NW	0.47 ± 0.07	1.5 ± 0.09	6.55 ± 0.07	0.05 ± 0.01	0.93 ± 0.27	14.84 ± 0.36	0.06 ± 0.02	0.9 ± 0.17	1.21 ± 0.2	6.93 ± 0.17	0.1 ± 0.02
propBA	1.51 ± 0.18	0.95 ± 0.18	30.39 ± 0.23	0.05 ± 0.01	1.26 ± 0.41	27.29 ± 0.58	0.04 ± 0.01	1.83 ± 0.33	1.7 ± 0.44	25.85 ± 0.47	0.06 ± 0.01
propW	0.41 ± 0.1	0.65 ± 0.17	24.24 ± 0.23	0.02 ± 0	1.34 ± 0.63	43.8 ± 1	0.03 ± 0.01	0.54 ± 0.22	1.74 ± 0.46	20.89 ± 0.49	0.02 ± 0.01
GL	0.7 ± 0.03	0.14 ± 0.02	1.06 ± 0.01	0.37 ± 0.01	0.93 ± 0.1	2.23 ± 0.08	0.29 ± 0.03	0.54 ± 0.04	0.37 ± 0.03	0.75 ± 0.01	0.33 ± 0.02
IWI	1.66 ± 0.32	6.33 ± 0.46	51.73 ± 0.47	0.03 ± 0.01	4.2 ± 1.97	152.01 ± 3.38	0.03 ± 0.01	2.36 ± 0.03	0.0001 ± 0	43.04 ± 0.55	0.05 ± 0

^a^TNB: total number of piglets born, NBA: number of piglets born alive, MUM: number of piglets born mummified, NSB: number of piglets stillborn, NBD: number of piglets born dead, NW: number of piglets weaned, propBA: proportion of piglets born alive, propW: proportion of piglets weaned, GL: gestation length, IWI: interval weaning to first insemination.

^b^propBA and propW were logit-transformed.

$\:{\varvec{\sigma\:}}_{\varvec{a}}^{2}$: genetic variance estimate

$\:{\varvec{\sigma\:}}_{\varvec{p}\varvec{e}}^{2}$: permanent environment variance estimate

$\:{\varvec{\sigma\:}}_{\varvec{e}}^{2}$: residual variance estimate

$$\:{\varvec{h}}^{2}$$: heritability estimate

For the combined analyses, heritability estimates for NSB, NBD, NW, and propBA were highest after the PRRS outbreak, mostly driven by a higher genetic variance and/or lower residual variance for these traits after the outbreak than before or during the outbreak (Table 6). Heritability estimates for NBA and MUM were highest during the PRRS break (Table 6).

Estimates of genetic correlations between phases for the combined purebred and crossbred data are reported in Table 7. Genetic correlation estimates for a given trait between phases were 0.03 to 0.59 units higher than estimates obtained for purebred sows and tended to have higher standard errors (Tables 4 and 7). Moderate genetic correlations were estimated for most traits, ranging from 0.23 to 0.85, expect for GL and propW. Strong, positive genetic correlations between phases were estimated for GL, ranging from 0.88 to 0.94 (Table 7). In contrast, weak, positive genetic correlations were estimated between phases for propW, ranging from 0.12 to 0.23. Standard errors were ≤ 0.19 for all traits, except for IWI and propW, for which standard errors ranged from 0.17 to 0.36 (Table 7).

Genetic correlations between different traits across phases were also estimated in the combined purebred and crossbred data, but the results were not presented due to convergence issues. This was likely due to the limited sample size and family data structure, indicating that larger datasets are needed for such analyses.

**Table 7:** Estimates of genetic correlations for reproductive performance traits between outbreak phases (before vs. during vs. after a PRRS outbreak) for the combined purebred and crossbred data

Trait^a^	Before vs. during	Before vs. after	During vs. after
TNB	0.70 ± 0.09	0.85 ± 0.05	0.80 ± 0.08
NBA	0.51 ± 0.11	0.80 ± 0.07	0.51 ± 0.13
NSB	0.74 ± 0.11	0.78 ± 0.06	0.77 ± 0.10
MUM	0.32 ± 0.15	0.69 ± 0.10	0.58 ± 0.13
NBD	0.59 ± 0.12	0.80 ± 0.06	0.51 ± 0.12
NW	0.45 ± 0.19	0.43 ± 0.13	0.48 ± 0.15
propBA^b^	0.57 ± 0.16	0.80 ± 0.09	0.63 ± 0.14
propW^b^	0.12 ± 0.33	0.12 ± 0.33	0.23 ± 0.33
GL	0.88 ± 0.04	0.94 ± 0.02	0.88 ± 0.03
IWI	0.23 ± 0.36	0.52 ± 0.17	0.54 ± 0.30

^a^TNB: total number of piglets born, NBA: number of piglets born alive, MUM: number of piglets born mummified, NSB: number of piglets stillborn, NBD: number of piglets born dead, NW: number of piglets weaned, propBA: proportion of piglets born alive, propW: proportion of piglets weaned, GL: gestation length, IWI: interval from weaning to first insemination.

^b^propBA and propW were logit-transformed.

### Animal reranking

The impact of PRRS on reranking of purebred animals based on GEBV was also evaluated. Table 8 presents Spearman rank correlations and Table 9 presents the proportion of animals that were selected in both phases for a given proportion selected. Similar correlations were observed for TNB, NSB, and GL across phases (Table 8). The lowest rank correlations were observed for propW, with values of -0.11, for purebred data and − 0.03 for the combined data (Table 8). The highest percentage of animals selected across phases was observed for GL, at 55.7% for the purebred data and 55.2% for the combined data (Table 9). In general, NW and propW had the lowest percentage of animals selected across phases and data used.

**Table 8:** Spearman rank correlations for genomic estimated breeding values for traits measured across outbreak phases based on analysis of the purebred and the combined purebred/crossbred data

Trait^a^	Before x during	Before x after	During x after
Only purebred data			
TNB	0.50	0.54	0.54
NBA	0.27	0.53	0.33
NSB	0.55	0.56	0.56
MUM	0.37	0.37	0.22
NBD	0.39	0.53	0.32
NW	0.16	0.14	0.16
propBA^b^	0.48	0.62	0.49
propW^b^	-0.11	0.18	0.37
GL	0.59	0.63	0.60
IWI	0.17	0.33	0.24
Purebred and crossbred data combined			
TNB	0.47	0.50	0.49
NBA	0.22	0.48	0.32
NSB	0.53	0.54	0.58
MUM	0.31	0.32	0.17
NBD	0.36	0.50	0.34
NW	0.14	0.15	0.20
propBA^b^	0.48	0.62	0.49
propW^b^	-0.03	0.17	0.52
GL	0.58	0.66	0.65
IWI	0.11	0.47	0.16

^a^TNB: total number of piglets born, NBA: number of piglets born alive, MUM: number of piglets born mummified, NSB: number of piglets stillborn, NBD: number of piglets born dead, NW: number of piglets weaned, propBA: proportion of piglets born alive, propW: proportion of piglets weaned, GL: gestation length, IWI: interval from weaning to first insemination.

^b^propBA and propW were logit-transformed

**Table 9:** Percentage of purebred animals selected across pairs of PRRS outbreak periods, for different proportions selected (1 to 20%) and use of purebred or combined purebred and crossbred data

Trait	Before vs. during				Before vs. after				During vs. after			
	1%	5%	10%	20%	1%	5%	10%	20%	1%	5%	10%	20%
Only purebred data												
TNB	7.14	26.19	42.48	49.05	9.52	31.43	38.90	48.21	16.67	28.10	40.57	50.36
NBA	0.00	9.09	19.66	32.15	11.43	26.70	35.04	46.66	2.86	9.09	16.24	29.30
NSB	14.71	34.91	43.49	49.63	20.59	28.40	32.25	45.63	14.71	31.95	35.80	46.67
MUM	8.82	20.93	31.40	40.03	2.94	11.63	18.02	39.01	5.88	17.44	19.48	28.82
NBD	24.24	34.97	44.17	46.24	18.18	23.93	30.98	46.54	12.12	15.95	23.31	37.48
NW	0.00	10.96	16.10	26.67	0.00	6.85	14.38	25.13	0.00	6.16	13.01	24.79
propBA^b^	0.00	15.79	35.37	46.51	17.39	37.72	42.36	50.87	0.00	8.77	20.96	39.96
propW^b^	0.00	1.35	6.04	15.77	0.00	2.70	12.08	30.20	20.00	17.57	40.27	45.64
GL	23.08	33.72	39.92	49.76	23.08	38.76	44.96	55.66	17.31	34.11	42.83	51.60
IWI	25.00	22.22	19.44	31.94	25.00	16.67	30.56	36.11	25.00	16.67	27.78	30.56
Purebred and crossbred data combined												
TNB	8.00	29.32	41.16	47.04	8.00	25.30	32.93	44.32	10.00	28.11	35.54	45.73
NBA	0.00	8.48	18.34	30.43	8.89	17.41	30.43	43.06	4.44	13.39	19.02	32.10
NSB	9.52	30.95	36.10	46.49	26.19	29.52	34.44	46.49	9.52	26.67	34.92	43.76
MUM	9.52	13.33	29.76	37.98	2.38	12.38	17.86	35.12	4.76	10.48	15.95	25.12
NBD	22.50	33.83	38.96	43.48	17.50	23.88	33.00	43.48	10.00	13.93	23.08	35.65
NW	0.00	5.59	15.73	29.20	3.45	9.09	14.34	27.10	3.45	6.99	14.69	26.40
propBA^b^	3.85	15.50	36.05	47.18	19.23	43.41	44.19	53.79	0.00	8.53	22.87	40.00
propW^b^	0.00	8.33	16.49	25.39	0.00	6.25	16.49	34.20	10.00	27.08	53.61	53.37
GL	18.97	29.55	36.77	48.80	24.14	32.99	44.16	55.15	22.41	38.83	45.36	54.81
IWI	11.11	20.93	18.39	19.08	33.33	18.60	32.18	47.98	11.11	16.28	17.24	24.86

^a^TNB: total number of piglets born, NBA: number of piglets born alive, MUM: number of piglets born mummified, NSB: number of piglets stillborn, NBD: number of piglets born dead, NW: number of piglets weaned, propBA: proportion of piglets born alive, propW: proportion of piglets weaned, GL: gestation length, IWI: interval weaning to first insemination.

^b^propBA and propW were logit-transformed

## Discussion

### Outbreak detection

PRRS outbreak periods were detected by observing declines in farrowing farm-year-week- estimates for reproductive performance from longitudinal data. As described by Zimmerman et al. [9], PRRS exposure can be divided into different phases, where the acute infection phase follows exposure and can last for two or more weeks, when the virus quickly spreads on the farm [9]. The second phase is characterized by reproductive failure, including increased piglet losses and pre-weaning mortality [9]. Notably, reproductive failure is often the first clinical sign reported on farms [9]. The third phase begins once reproductive performance stabilizes to pre-outbreak levels, although PRRSV may persist within the herd [8]. The period after an outbreak represents the endemic phase, during which pigs may remain infected for several months [8, 9]. The PRRS outbreak periods detected using longitudinal data analyses were consistent with the PRRS outbreak periods reported by each farm through diagnostic laboratory testing and showed overlapping timing between purebred and crossbred populations on the same farms, confirming the accuracy of the detection method used in this study. Several studies have applied similar approaches to detect PRRS outbreak periods using phenotypic time series data. For example, Lewis et al. [25] evaluated 30-day rolling averages of mummified piglets per litter and Serão et al. [23] applied similar methods to Landrace sows using trends in NBA, piglets alive at 24 h, NSB, and MUM. Estimates of farrowing year-week effects have also been used in Canadian commercial herds [26], in Large White × Landrace sows [24], and in purebred Yorkshire and Landrace populations [21, 22].

The results obtained from this study align with findings from previous research [21, 23–26], which reported lower intrauterine and pre-weaning piglet survival during a PRRS outbreak, indicated by lower NBA, NW, propBA, and propW, and higher NSB, MUM, and NBD. A decrease in GL was also observed during outbreaks for this study, indicating the occurrence of premature farrowing due to PRRSV-infection, as previously reported [30]. We also observed an increase in the farrowing-year-week estimates for IWI in purebred sows (Fig. 1), along with higher IWI during the outbreaks (Fig. 3). These findings align with results from previous studies, which reported that PRRS may delay return to estrus [31, 32]. After the acute infection phase, reproductive performance and pre-weaning mortality typically return to pre-exposure levels [9], as shown for the farms analyzed in this study.

### Genetic parameters across disease phases

For most previous studies, PRRS data were split in two phases, with data from the pre- and post-outbreak phases combined [24–26]. However, PRRS may persist within a herd for months following initial exposure [9, 33]. Therefore, for this study, data were analyzed in three phases: before, during, and after PRRS exposure. Evaluating performance months after the acute infection phase enabled evaluation of the long-term impact of PRRSV-infection on reproductive performance, which may not be realized when analyzing disease data using a two-phase approach [34].

Of the three disease outbreak phases, genetic and residual variance component estimates were highest during and after a PRRS outbreak. These results agree with findings from previous studies, which report higher variance component estimates during vs. before an outbreak [21, 22, 24]. Results reported by Berghof et al. [10] suggest that the presence of environmental stressors, such as disease, increases genetic variation in micro-environmental sensitivity. Interestingly, when combining data from purebred and crossbred sows, the estimates of residual variance for a given trait measured after the outbreak were more similar to those obtained before vs. during an outbreak. Standard errors for variance components and heritability estimates were higher during the outbreak period, as expected, due to fewer records available for those periods in comparison to before and after the outbreak.

Our heritability estimates for traits measured during the pre-PRRS phase align with those reported for these same traits measured under PRRSV-free conditions in previous studies [35–41]. Heritability estimates observed across the PRRS outbreak phases followed a similar trend to previous studies, where heritability was typically estimated to be higher for litter size traits (i.e., TNB, NBA, NSB, MUM, NBD, and NW) measured during a PRRS outbreak vs. in PRRSV-free conditions [21, 22, 24–26], driven by increased genetic variance during the outbreak phase. Increased genetic variance and higher heritability estimates during the outbreak phase highlight genetic variation in host response to PRRS challenge [13]. Hickmann et al. [22] also reported higher heritability estimates for TNB, NSB, MUM, NBD, and NW for the post-PRRS phase for Duroc sows, and for TNB, NBD, and NSB for Landrace sows [42, 43]. Higher genetic variance and heritability estimates after an outbreak may reflect underlying genetic variation in resilience to persistent PRRSV-infection [42]. Ongoing exposure after the acute outbreak, coupled with the rise in residual variance during the outbreak and its decline afterwards, may enhance the expression and detection of additive genetic variation related to PRRS resilience.

The traits propBA and propW are indicators of intrauterine and pre-weaning survival, respectively. Heritability of piglet survival is typically low, ranging from 0.02 to 0.10 [44–46], which is consistent with our estimates obtained for propBA and propW for the purebred data and the combined purebred and crossbred data, but not of the crossbred data. Scanlan et al. [24] estimated that heritability for the proportion of piglets born dead (1 – propBA) was higher in a PRRS-challenged vs. a PRRSV-free environment. It is important to note, however, that propBA and propW are ratio traits, which, although easy to interpret, are rarely normally distributed, with deviations from normality increases as the coefficient of variation increases [47], which was addressed using a logit transformation. Predicting response to direct selection for ratio traits is also challenging, as the trait with a higher genetic variance tends to receive disproportionate selection pressure [48]. Moreover, ratio traits are strongly correlated with their component traits. Studies also indicate that direct selection for ratio traits typically results in less genetic gain compared to alternative approaches, such as using a linear selection index of the component traits designed to reduce the numerator traitand increases the denominator trait [49].

This is the first study to evaluate the impact of a PRRS outbreak on genetic parameters for GL and IWI. While PRRS exposure influenced variance components for GL, the estimates of genetic variance was less affected for GL compared to other traits. Additionally, heritability estimates for GL across all phases (ranging from 0.28 to 0.45) align with previous reports (ranging from 0.21 to 0.39) for purebred Landrace and Large White pigs under PRRSV-free conditions [39–41]. Variance component estimates for IWI were highest during the outbreak, although heritability estimates remained low (ranging from 0.02 to 0.04) across all outbreak phases, which agrees with heritability estimates reported in the literature for Landrace pigs under PRRSV-free conditions (ranging from 0.02 to 0.08) [35, 37].

Sows with superior reproductive performance under a PRRS-challenge are also expected to have superior immune response to PRRSV-infection [21–26]. Serão et al. [23] demonstrated that antibody response to PRRSV-infection, measured as the sample-to-positive (S/P) ratio, is genetically correlated with reproductive traits during a PRRS outbreak with estimates ranging from − 0.72 to 0.73. Similarly, Hickmann et al. [50] reported favorable genetic correlations of S/P ratio with NBA, NBD, and NSB during the outbreak phase, ranging from − 0.24 to 0.30 in Duroc pigs and from − 0.33 to 0.61 in Landrace pigs. Hickmann et al. [50] also identified significant genomic regions associated with S/P ratio linked to reproductive performance traits recorded outside of the PRRS outbreak period. Laplana et al. [42] identified genomic regions associated with stability of reproductive performance during a PRRS outbreak, including genes related to viral entry and immune response. Abella et al. [51] proposed that sows could be classified as resilient to future reproductive challenges based on their immune response to vaccination using a modified-live PRRSV vaccine. Fraile et al. [52] later validated this phenotyping method, demonstrating that vaccination may be used to identify sows with better reproductive performance during and after a PRRS outbreak.

### Purebred-crossbred genetic correlations

Crossing purebred populations to produce commercial animals is a standard practice in the pig breeding industry. These breeding programs aim to improve crossbred performance on commercial farms by selecting purebred animals, often based on purebred performance. Consequently, the accuracy of selection depends on the genetic correlation between purebred and crossbred performance [53, 54]. In this study, high genetic correlations between purebred and crossbred animals were estimated for most traits, suggesting that some indicators of reproductive performance of purebred and crossbred sows are likely influenced by the same genetic variants with similar effects [53], however, potential scale effects between populations may also contribute to the magnitude of these correlations. This supports the approach of combining purebred and crossbred performance data for variance component estimation and genetic evaluation of some reproductive performance traits that appear to have a similargenetic basis in purebreds and crossbreds.

It is well established that r_pc_ estimates tend to decrease when purebred and crossbred animals are raised in contrasting environments, such as nucleus versus commercial farms [53]. Nucleus farms are typically highly controlled, with strict biosecurity, small-group or individual housing, and specialized feeding. In contrast, animals reared at commercial farms are exposed to more variable conditions, including increased risk of pathogen exposure [55]. In this study, data were collected from multiplier farms, which have relatively similar management and environmental conditions across farms and represent an intermediate production level between nucleus and commercial herds. Both farms with crossbred animals also had purebred animals which were included in the analyses. These factors likely contributed to the high r_pc_ estimates (> 0.90) observed for most traits (TNB, NBA, NSB, NBD, NW, and GL). However, low-to-moderate r_pc_ estimates were obtained for MUM, propBA, propW, and IWI, ranging from 0.11 to 0.77. These deviations may be explained by dominance and epistatic effects not accounted for in the model, differences in allele frequencies between purebred and crossbred populations, differences in allele substitution effects according to the breed of origin, and genotype-by-environment (G×E) interactions resulting from environmental differences between purebred and crossbred rearing systems, even within a farm [53, 54].

### Genotype-by-environment interactions for reproductive performance between PRRS outbreak phases

Genotype-by-environment interactions can be assessed using multi-trait models, where a given trait is analyzed as distinct traits when measured in different environments [56]. This allows for the detection of potential changes in animal performance, due to environmental challenges. A genetic correlation of “1” for the same trait measured in different environments indicates a lack of GxE, whereas estimates below 0.80 suggest strong GxE interactions and potential reranking of animals based on EBV for different environmental conditions [57, 58]. In this study, GxE was evaluated across PRRS outbreak phases by estimating genetic correlations for a given reproductive performance trait before vs. during vs. after the PRRS outbreak. Our estimates of genetic correlations for traits measured before and during the PRRS outbreak align with those reported in previous studies for reproductive performance evaluated in the absence and presence of PRRS. Reported estimates range from 0.32 to 0.96 for TNB, from 0.21 to 0.98 for NBA, from 0.33 to 0.83 for NSB, from − 0.13 to 0.90 for NBD, and from 0.27 to 0.94 for NW [21, 22, 24–26].

Our estimates of genetic correlations for reproductive performance traits measured across disease phases ranged from 0.09 to 0.99, with most of traits having moderate-to-strong (> 0.30) genetic correlations estimates. These results indicate that reproductive performance under disease challenge can be improved by selecting animals based on reproductive performance measured in a PRRSV-free environment. Genetic correlation estimates were generally higher between the before and after PRRS outbreak phases, compared to between the before and during or the during and after outbreak phases. These results indicate that reproductive performance before and after an outbreak are genetically more similar than reproductive performance before vs. during and during vs. after an outbreak. This also suggests that reproductive performance of PRRS naïve sows and reproductive performance of sows that have recovered from PRRSV-infection may have a more similar genetic basis [22]. However, G×E was still evident between the phases before and after the outbreak, as indicated by the genetic correlations significantly lower than 1 for almost all traits, leading to reranking of animals. This suggests that top-performing individuals in a PRRSV-free environment may not necessarily be the top-performing individuals during or after an outbreak.

Estimates of genetic correlations between PRRS outbreak phases were lower when using combined purebred and crossbred data compared to only purebred data, suggesting that GxE is more pronounced when crossbred performance is also considered [53, 55]. The lower correlations observed in the combined dataset likely reflect greater environmental heterogeneity and possible genetic scale differences in the crossbred populations, which may amplify interaction effects across outbreak phases. These results indicate that accounting for GxE between PRRSV-free vs. PRRSV-challenged environments, as well as between purebred vs. crossbred performance, is critical to maximize response to selection for improved PRRS resilience.

A very impactful effect of GxE is the reranking of animals in different environments [58]. Therefore, reranking of animals between different outbreak phases was evaluated using Spearman rank correlation of GEBV and by examining the percentage of purebred animals that would selected regardless of PRRS outbreak phase data used, and when including only purebred data vs. purebred and crossbred data. Results revealed significant reranking of animals, with a low proportion of top-performing animals in one phase maintained superior performance in other phases. These findings suggest that the top-performing animals in a PRRSV-free environment may not necessarily be the top-performing animals during or after a PRRS outbreak.

### Implications and limitations

Previous studies have demonstrated genetic variation in reproductive performance across PRRS outbreak phases [21, 22, 24–26], but these analyses were limited to using data from either purebred [21–23] or crossbred [23] populations. Data on both purebred and crossbred sows were used in the current study, including purebred and crossbred sows reared at the same farm. Results show that reproductive performance during and after a PRRS outbreak can be improved by selecting animals based on data recorded in a PRRSV-free environment. However, noticeableGxE interactions were observed for reproductive performance measured in a PRRSV-free environment (before the outbreak) with reproductive performance measured during and after an outbreak. Therefore, collecting data on animals post-PRRSV exposure, particularly on crossbred sows, is expected to improve selection for enhanced disease resilience of crossbreds in commercial environments.

Proper recording of cross-fostering is essential when evaluating female reproductive and maternal ability traits. Unfortunately, this information was not comprehensively recorded in the studied herds. Access to this data would have enabled more accurate evaluation of the number of biologicalpiglets weaned and the total number of piglets weaned by the sow (including both biological and non-biological offspring). Access to cross-fostering data is also expected to improve evaluation of propW, which indicates pre-weaning survival rates.

The traits IWI and propW had estimates of heritabilitywith high standard errors. Therefore, access to larger datasets for these traits is needed to improve genetic parameter estimation. Genotype data was available for the sires of purebred sows, but not the sows themselves. Including genotypes for purebred sows would have been beneficial for obtaining more accurate estimates of genetic parameters and GEBVs across PRRS outbreak phases. Pedigree information was only available for one of the two farms with crossbred sows, limiting the ability to link purebred and crossbred animals. Moreover, only a small proportion (about 20%) of crossbred animals with recorded phenotypes were genotyped. Expanding the number of animals with both genotypic and phenotypic data across the different PRRS outbreak phases would improve the accuracy of GEBVs and of estimates of genetic parameters.

## Conclusions

PRRS outbreak periods can be identified based on herd-level variation in longitudinal reproductive performance data. Our findings confirm that reproductive performance is heritable before, during, and after a PRRS outbreak in both purebred (Large White) and crossbred (Landrace x Large White) populations. However, additive genetic variance differs between these outbreak phases, with higher variance during the outbreak. Genetic correlation analyses indicated that reproductive performance traits may not be influenced by the same set of genes across outbreak phases, or that the genes influencing these traits have different effects under different conditions (i.e., GxE). These interaction effects are more pronounced when combining purebred and crossbred data, which was demonstrated by the lower genetic correlation estimates for the same trait across outbreak phases in the combined versus the purebred data. Additionally, the presence of GxE across outbreak phases indicate that the top-performing individuals in a PRRSV-free environment are not necessarily the top-performers in a PRRSV-challenged environment. However, favorable genetic correlations across phases suggest that selection for improved reproductive performance (using data collected under non-challenged conditions) also improves reproductive performance under PRRS challenged conditions. Nevertheless, incorporating data from sows following PRRSV-exposure and including purebred and crossbred information is expected to capture additional genetic variation in response to PRRS and, ultimately, aid in increased PRRS resilience of crossbred sows in commercial environments.

## Supplementary Information

Below is the link to the electronic supplementary material.

Supplementary Material 1

Supplementary Material 2

## Data Availability

All the data and materials needed for the interpretation of the results are presented in the paper and its supplementary material. The raw datasets cannot be made publicly available because they are owned by swine producers and this information is commercially sensitive.
