# Supplementary material for: Genetics of reproductive performance across Porcine Reproductive and Respiratory Syndrome (PRRS) outbreak phases in purebred and crossbred sows
Source: Genet Sel Evol. 2025 Oct 30;57:62. doi: 10.1186/s12711-025-01011-y (PMC12577220; doi:10.1186/s12711-025-01011-y)
Supplement: Supplementary file 1 — Supplementary Material 1 [file 12711_2025_1011_MOESM1_ESM.docx]

**Supplementary Table S1.** Timing and duration of PRRS outbreaks, detected for each farm and sow population.

| **Purebred Large White** | | | |
| --- | --- | --- | --- |
| **Farm** | **Start** | **End** | **Duration (days)** |
| Farm 1 | 20 November 2022 | 24 April 2023 | 155 |
| Farm 2 | 18 December 2022 | 27 March 2023 | 99 |
| Farm 3 | 02 April 2023 | 11 September 2023 | 162 |
| Farm 4 | 16 April 2023 | 14 August 2023 | 120 |
| **Crossbred (Landrace x Large White)** | | | |
| **Farm** | **Start** | **End** | **Duration (days)** |
| Farm 1 | 20 November 2022 | 09 April 2023 | 140 |
| Farm 4 | 02 April 2023 | 21 August 2023 | 141 |
